# Supplementary material for: Shift of Aromatic Profile in Probiotic Hemp Drink Formulations: A Metabolomic Approach
Source: Microorganisms. 2019 Oct 29;7(11):509. doi: 10.3390/microorganisms7110509 (PMC6920803; doi:10.3390/microorganisms7110509)
Supplement: Supplementary file 1 [file microorganisms-07-00509-s001.zip › supplementary table 1.docx]

Table S1: Microbial quantification, pH values and nutritional label values.

|  | **[∆ pH t6]^1^** | **[∆ pH t24]^2^** | **∆ growth t6^3^** | **∆ growth t24^4^** | **Energy^5^** | **Proteins^6^** | **Fats^6^** | **Carbohydrates^6,7^** | **Fiber^6^** | **Salt^6^** |
| --- | --- | --- | --- | --- | --- | --- | --- | --- | --- | --- |
| Hlp | 2.06 | 3 | 0.29 | 1.51 | 0.4 | 1.0 | 2.6 | 0.2 | 1 | 0.003 |
| Hlf | 0.92 | 2.47 | 0.29 | 1.53 | 0.4 | 1.0 | 2.6 | 0.2 | 1 | 0.003 |
| Hbb | 1.71 | 2.56 | 0.78 | 1.98 | 0.4 | 1.0 | 2.6 | 0.2 | 1 | 0.003 |
| Hc | 1.78 | 2.88 | 0.55 | 1.78 | 0.4 | 1.0 | 2.6 | 0.2 | 1 | 0.003 |
| Slp | 1.66 | 3.55 | 1.03 | 2.99 | 0.36 | 3 | 1.7 | 0.5 | 0.5 | 0.05 |
| Slf | 0.68 | 3.29 | 1.36 | 3.31 | 0.36 | 3 | 1.7 | 0.5 | 0.5 | 0.05 |
| Sbb | 0.02 | 3.3 | 1.66 | 3.62 | 0.36 | 3 | 1.7 | 0.5 | 0.5 | 0.05 |
| Sc | 1.3 | 3.55 | 1.18 | 3.14 | 0.36 | 3 | 1.7 | 0.5 | 0.5 | 0.05 |
| Rlp | 2.44 | 3.74 | 0.85 | 3.03 | 0.64 | 0.6 | 2 | 8 | 0.2 | 0.08 |
| Rlf | 1.44 | 3.84 | 1.04 | 3.23 | 0.64 | 0.6 | 2 | 8 | 0.2 | 0.08 |
| Rbb | 0.32 | 3.77 | 1.17 | 3.63 | 0.64 | 0.6 | 2 | 8 | 0.2 | 0.08 |
| Rc | 2.15 | 3.79 | 0.83 | 3.02 | 0.64 | 0.6 | 2 | 8 | 0.2 | 0.08 |
| HRlp | 1.495 | 2.825 | 1.12 | 2.31 | 0.52 | 0.8 | 2.3 | 4.1 | 0.6 | 0.0415 |
| HRlf | 0.415 | 2.455 | 1.17 | 2.23 | 0.52 | 0.8 | 2.3 | 4.1 | 0.6 | 0.0415 |
| HRbb | 1.8 | 2.675 | 1.63 | 4.52 | 0.52 | 0.8 | 2.3 | 4.1 | 0.6 | 0.0415 |
| HRc | 1.75 | 2.85 | 2.06 | 4.95 | 0.52 | 0.8 | 2.3 | 4.1 | 0.6 | 0.0415 |
| HSlp | 1.6 | 3.745 | 1.53 | 4.02 | 0.38 | 2.0 | 2.15 | 0.7 | 0.75 | 0.0265 |
| HSlf | 0.36 | 4.095 | 1.06 | 3.75 | 0.38 | 2.0 | 2.15 | 0.7 | 0.75 | 0.0265 |
| HSbb | 1.97 | 3.15 | 1.11 | 4 | 0.38 | 2.0 | 2.15 | 0.7 | 0.75 | 0.0265 |
| HSc | 1.56 | 4.045 | 1.86 | 5.05 | 0.38 | 2.0 | 2.15 | 0.7 | 0.75 | 0.0265 |
| SRlp | 2.31 | 3.81 | 1.29 | 3.48 | 0.5 | 1.8 | 1.85 | 4.25 | 0.35 | 0.065 |
| SRlf | 1.595 | 3.79 | 1.15 | 3.64 | 0.5 | 1.8 | 1.85 | 4.25 | 0.35 | 0.065 |
| SRbb | 0.53 | 3.86 | 1.08 | 3.97 | 0.5 | 1.8 | 1.85 | 4.25 | 0.35 | 0.065 |
| SRc | 2.07 | 3.85 | 1.24 | 3.42 | 0.5 | 1.8 | 1.85 | 4.25 | 0.35 | 0.065 |

^1^mean difference of pH values between 0 and 6 hours, expressed as an absolute value; ^2^mean difference of pH values between 0 and 24 hours, expressed as an absolute value; ^3^mean difference of growth between 0 and 6 hours, expressed in Log10 cells/ml; ^4^mean difference of growth between 0 and 24 hours, expressed in Log10 cells/ml; ^5^Kcal/g, obtained by nutritional label; ^6^g/100g, obtained from nutritional labels; ^7^reported as sugars.
