# Supplementary material for: Shift of Aromatic Profile in Probiotic Hemp Drink Formulations: A Metabolomic Approach
Source: Microorganisms. 2019 Oct 29;7(11):509. doi: 10.3390/microorganisms7110509 (PMC6920803; doi:10.3390/microorganisms7110509)
Supplement: Supplementary file 1 [file microorganisms-07-00509-s001.zip › supplementary table 2.docx]

Table S2. VOCs quantitation of non-fermented drinks.

| **Compound** | **H** | **R** | **S** | **H/R** | **H/S** | **S/R** |
| --- | --- | --- | --- | --- | --- | --- |
| cyclopropanemethanol | tr. | tr. | n.d. | tr. | tr. | tr. |
| 2-hexanol | n.d. | n.d. | tr. | tr. | tr. | tr. |
| hexanol, 2-ethyl | tr. | n.d. | n.d. | n.d. | tr. | n.d. |
| heptanol | n.d. | n.d. | tr. | n.d. | tr. | n.d. |
| 2-heptanol, 2-methyl | + | n.d. | + | tr. | tr. | tr. |
| octanol | n.d. | n.d. | tr. | n.d. | tr. | n.d. |
| 1-octen-3-ol | tr. | n.d. | n.d. | tr. | tr. | n.d. |
| acetic acid | ++ | + | + | ++ | + | + |
| propanoic acid | + | n.d. | n.d. | + | + | n.d. |
| propanoic acid, 3-methyl | n.d. | n.d. | n.d. | + | ++ | n.d. |
| butanoic acid | tr. | n.d. | n.d. | + | ++ | tr. |
| pentanoic acid | + | n.d. | n.d. | + | + | n.d. |
| hexanoic acid | + | n.d. | n.d. | ++ | ++ | n.d. |
| heptanoic acid | + | n.d. | n.d. | + | + | n.d. |
| octanoic acid | + | n.d. | n.d. | + | ++ | n.d. |
| nonanoic acid | tr. | + | + | + | + | + |
| butanal, 3-methyl | n.d. | + | + | n.d. | n.d. | + |
| furfural | + | + | n.d. | n.d. | n.d. | tr. |
| hexanal | +++ | ++ | +++ | + | ++ | ++ |
| 2-hexenal (E) | n.d. | tr. | + | n.d. | n.d. | n.d. |
| heptanal | ++ | ++ | + | ++ | + | tr. |
| 2-heptenal (Z) | ++ | + | + | ++ | ++ | tr. |
| 2,4-heptadienal | + | n.d. | tr. | tr. | tr. | n.d. |
| octanal | n.d. | ++ | ++ | n.d. | + | n.d. |
| nonanal | n.d. | ++ | ++ | + | + | + |
| decanal | + | + | tr. | tr. | + | tr. |
| benzaldehyde | + | + | ++ | ++ | + | + |
| benzaldehyde, 3,4-dimethyl | + | n.d. | n.d. | tr. | tr. | n.d. |
| furan, 2-methyl | tr. | tr. | + | tr. | tr. | tr. |
| cyclohexane, 1,1,3,5-tetramethyl | n.d. | + | n.d. | tr. | tr. | n.d. |
| cycloheptane | + | n.d. | tr. | n.d. | n.d. | tr. |
| heptane, 2,4-dimethyl | ++ | n.d. | tr. | n.d. | n.d. | tr. |
| octane, 2-methyl | + | n.d. | n.d. | tr. | tr. | tr. |
| octane, 2,6-dimethyl | + | n.d. | + | tr. | tr. | + |
| octane, 1-chloro | n.d. | + | + | tr. | n.d. | tr. |
| 2-octene (Z) | n.d. | n.d. | tr. | tr. | n.d. | tr. |
| acetone | tr. | tr. | ++ | + | + | ++ |
| 2-butanone | + | n.d. | n.d. | tr. | tr. | n.d. |
| 2-pentanone | n.d. | n.d. | n.d. | + | + | tr. |
| 3-penten-2-one, 3-methyl | n.d. | ++ | ++ | + | tr. | ++ |
| 3-hexanone, -methyl | n.d. | ++ | + | + | + | + |
| 2-heptanone | n.d. | + | + | n.d. | n.d. | + |
| 2-heptanone, 4-methyl | + | ++ | + | + | + | + |
| 2-octanone | + | n.d. | + | n.d. | n.d. | n.d. |
| 2-nonanone | n.d. | + | tr. | tr. | n.d. | tr. |
| 2-decanone | n.d. | + | tr. | tr. | n.d. | tr. |

N.d.: not detected; Tr.: traces (≤ 0.05); + ≤ 0.3: ++ = 0.3 ÷ 0.7; +++ = 0.7 ÷ 1.

Normalized data with the mean centering method. H = mean of hemp drinks; S = mean of soy drinks; R = mean of rice drinks; HR = mean of hemp/rice drinks; HS = mean od hemp/soy drink; SR = mean of soy/rice drinks. Data were obtained from duplicate samples and two independent experiments.
